# Use of Tangible, Educational, and Psychological Supportive Services Among Chinese American Dementia Caregivers

**DOI:** 10.1093/geroni/igab046.878

**Published:** 2021-12-17

**Authors:** Jinyu Liu, Yifan Lou, Ethan Siu Leung Cheung, Bei Wu

**Affiliations:** 1 Columbia University, Columbia University, New York, United States; 2 Columbia University, New York, New York, United States; 3 New York University, New York, New York, United States

## Abstract

Background and Objectives: Though many studies have examined the service utilization of dementia caregivers, there is limited empirical evidence from Asian Americans and the lack of incorporating community resources and sociocultural factors in this field. Guided by the Andersen's Behavioral Model of Health Services Use (ABM), we aimed to understand whether and how predisposing, enabling and need factors were associated with utilizing multiple types of services among Chinese Americans dementia caregivers. Research Design and Methods: We collected survey data from 134 Chinese dementia caregivers in New York City. Logistic regression models were conducted to test the associations between predisposing, enabling and need factors and the likelihoods of using tangible (home health aide, adult daycare, respite care), educational (lectures and workshops), and psychological (peer support groups and psychological counseling) services. Results: Consistent with prior literature, caregiver’s knowledge about services, caring tasks, length of care and burden and care recipient’s physical and cognitive deteriorations, were significantly associated with higher possibilities of using multiple types of services among these Chinese American dementia caregivers. Three sociocultural factors, including residing in Chinatowns, availability of alternative family caregivers and diagnosis of cognitive deterioration, were also associated with higher likelihoods of using educational or psychological services. Discussion and Implications: The findings extended the existing literature on service utilization of caregivers by highlighting the importance of distinguishing types of services and the necessity of considering sociocultural factors in future research and practice.

